# Do psychological factors or radiographic severity play a role in the age of onset in symptomatic developmental dysplasia of hip and femoroacetabular impingement syndrome?

**DOI:** 10.1186/s12891-019-2784-9

**Published:** 2019-09-05

**Authors:** Shawn Okpara, Paul Nakonezny, Joel Wells

**Affiliations:** 1 Department of Orthopaedic Surgery, University of Texas Southwestern Medical Center, 1801 Inwood Rd 1st floor, Dallas, TX 75390 USA; 2 Department of Population and Data Sciences, Division of Biostatistics, University of Texas Southwestern Medical Center, Dallas, USA

**Keywords:** Hip, Femoroacetabular impingement syndrome, Pain catastrophizing, Developmental dysplasia of hip

## Abstract

**Background:**

Age of onset in symptomatic developmental dysplasia of the hip (DDH) and femoroacetabular impingement syndrome (FAIS) varies. The purpose of this study was to investigate whether psychological factors, radiographic, and clinical variables were related to age of onset of hip pain in DDH and FAIS.

**Methods:**

We collected demographic, clinical, and radiographic data on 56 DDH and 84 FAIS patients. Each was diagnosed based on radiographic findings and clinical history. Age of onset was operationalized by subtracting patient reported duration of symptoms from patient age at presentation. Pain catastrophizing (PCS) and depression were assessed with the pain catastrophizing scale and hospital anxiety and depression scale (HADS), respectively. Multiple linear regression modeling, with Lasso variable selection, was implemented.

**Results:**

Pain catastrophizing, anxiety, and depression were not significantly related to age of DDH onset (*p*-values > 0.27) or age of FAIS onset (*p*-values > 0.29). LASSO-penalized linear regression revealed alpha Dunn angle, Tonnis grade, prior hip surgery, WOMAC pain score, and iHOT total score were associated with age of onset in FAIS (Adjusted *R*^2^ = 0.3099). Lateral center edge angle (LCEA), alpha frog angle, Tonnis grade, SF12 physical functioning, and body mass index (BMI) were associated with age of DDH onset (Adjusted *R*^2^ = 0.3578).

**Conclusions:**

Psychological factors, as measured by PCS and HADS, were not associated with age of onset in DDH or FAIS. Functional impairment as measured by WOMAC pain and impaired active lifestyle as measured by iHOT were found to affect age of FAIS onset. For DDH, impaired physical functioning and increasing BMI were found to be associated with age of onset. Severity of the disease, as measured radiographically by LCEA and alpha Dunn angle, was also found to be associated with earlier age of onset in DDH and FAIS, respectively. A patient’s radiographic severity may have more of a relationship to the onset of pain than physiologic factors.

## Introduction

Developmental dysplasia of the hip (DDH) and femoroacetabular impingement syndrome (FAIS) are two common hip pathologies that may lead to early onset of hip pain [1–4]. High activity levels are recognized as a common factor among young individuals diagnosed with FAIS [1–3, 5]. Similarly, risk factors such as increased activity level and severity of DDH, have been shown to correlate with a younger age of presentation for periacetabular osteotomy (PAO) in symptomatic DDH [6]. Despite this, age of onset for DDH or FAIS has not been studied in the context of an individual’s mental health status - specifically pain catastrophizing, anxiety, and depression.

Pain catastrophizing is defined as an exaggerated negative mental state during, or in anticipation of, a painful experience. The Pain Catastrophizing Scale (PCS) is often used to objectify exaggeration of pain. Pain catastrophizing modulation and the perception of pain can affect coping mechanisms and cause a patient to have increased pain for the same pathology [7, 8]. The mental health component may be more related to symptom severity than the degree of radiographic deformity in patients with FAI syndrome [9]. However, at present, we are unaware of the role of pain catastrophizing on the age of onset of pain in DDH and FAIS.

Depression and anxiety are psychiatric conditions known to have a negative impact on an individual’s well-being [10, 11]. Mental health has been established as a significant predictor of an individual’s quality of life after surgery [12]. In fact, greater depression scores have been shown to lead to worse reported outcomes in patients who pursued surgical treatment for DDH and FAIS [13–15]. Though, like pain catastrophizing, little is known about the role of depression and anxiety in determining the age of onset in this unique patient population.

Age of onset is an important variable in the young adult hip population. Age has been shown to be a negative predictor for treatment in patients undergoing treatment for DDH and FAI syndrome [15–18]. The primary aim of this study was to determine whether pain catastrophizing, anxiety, or depression are associated with age of onset in DDH and FAIS. A secondary aim was to assess whether other radiographic and clinical variables affect the age of onset in DDH and FAIS.

## Materials and methods

We evaluated all patients presenting to a specialist hip clinic with the main complaint of hip pain, at a single academic medical center (University of Texas Southwestern Medical Center, Dallas, Texas, United States). Institutional review board approval was obtained from the University of Texas Southwestern Medical School STU 122016–058, all patients gave written consent. The primary outcome was age of onset (years) for symptomatic DDH and FAIS. Age of onset was operationalized by subtracting patient reported duration of symptoms from patient age at presentation. This outcome variable has previously been used in prior hip pathology studies [16].

We evaluated 140 patients presenting to a comprehensive orthopedic hip clinic, with a chief complaint of hip pain, and who were diagnosed with either FAIS (*n* = 84) or DDH (*n* = 56), at the UT Southwestern academic medical center between November 2016 and April 2018. Once consented and enrolled in the study, all 140 patients were asked to complete an IRB-approved self-report hip questionnaire that included questions about their symptoms, activity level, general health (physical and mental), as well as a pain catastrophizing scale. Functional impairment, as measured by patient reported questionnaires (i.e SF12, WOMAC) represents decreased quality of life and increased limitations to activities of daily living (ADL) due to pain and symptoms [17]. This study was reviewed and approved by the institutional review board at the University of Texas Southwestern Medical Center.

All patients had a diagnosis of DDH or FAIS established by the treating orthopedic surgeon (JW) who was fellowship-trained in hip preservation. For DDH, patients who presented to the treating physician with symptomatic developmental dysplasia of the hip, radiographic evidence of femoral head uncovering, and a lateral center-edge angle on standard radiographs of < 25° were offered treatment with PAO and included in this study [3, 6]. Exclusion criteria were those with diagnosis other than DDH. For FAIS, inclusion criteria included patients with clinical symptoms or physical exam associated with FAI syndrome or imaging findings such as pincer or cam deformity and/or alpha angle greater than 55° and were offered surgical treatment for their FAIS [18, 19]. Exclusion criteria include patients that do not have a physical exam or radiologic findings associated with FAIS, and those found to have a different hip diagnosis.

An initial pool of 19 characteristic variables was selected for analysis as potential covariates of age of onset (Table 1). These variables were selected based on the results of previously published findings that have been associated with symptomatic DDH and FAIS. The pool of potential variables selected included: Sex, BMI (> 30 kg/m2 vs. ≤30 kg/m2) [6] history of hip surgery [6], laterality [6], Tonnis grade [20, 21], Tonnis angle [21], anterior center edge angle [6, 20, 21], lateral center edge angle [5, 15, 16], alpha Dunn angle [20, 21], alpha frog angle [5, 16], International Hip Outcome Tool (iHOT) total score [22], Hip Outcome Score [22, 23], the UCLA activity score [6, 23], Pain Catastrophizing Scale (PCS) Total score [7, 8, 24], SF-12 Physical Functioning (activity level) subscale score, SF-12 Physical Health subscale score [23], Hospital Anxiety and Depression Scale [25], Womac Total Score and Womac Pain subscale score [23]. Reliability and validity of these various scales have been previously established [17, 26–31]. Each of the variables was measured during the most recent clinic visit for the patient’s orthopedic hip complaint, which was on average 3.37 (SD = 5.30) years and 4.35 (SD = 4.07) years since the onset age for the patient’s symptomatic DDH and FAIS diagnosis, respectively.

**Table 1:** Variables tested in study

Variables Tested	
1. Sex (Male vs Female)	
2. BMI (> 30 kg/m^2^ vs. ≤30 kg/m^2^)	
3. Side of Lesion (Left vs Right)	
4. Prior Hip Surgery (Yes vs No)	
5. Tonnis Grade (Grade 1 vs. Grade 0)	
6. Tonnis Angle (°)	
7. Anterior Center Edge Angle (°)	
8. Lateral Center Edge Angle (°)	
9. Alpha Dunn Angle (°)	
10. Alpha Frog Angle (°)	
11. International Hip Outcome Tool (iHOT): 0–100; 0 = lowest quality of life; 100 = highest quality of life	
12. Hip Outcome Score (HOS): %0–100; %0 = greatest difficulty; %100 = lowest difficulty	
13. UCLA Activity Score: 0–10; 0 = no activity; 10 = greatest activity	
14. Pain Catastrophizing Score (PCS): 0–52; 0 = lowest level of catastrophizing; 52 = greatest level of catastrophizing	
15. SF-12 Physical Functioning Subscale Score: 0–100;0 = lowest physical functioning level; 100 = greatest physical functioning level	
16. SF-12 Physical Health Subscale Score:0–100;0 = lowest physical health; 100 = greatest physical health	
17. Hospital Anxiety and Depression Scale: 0–21; 0 = lowest level of anxiety/ depression; 21 = highest level of anxiety/depression	
18. WOMAC Pain Subscale Score: 0–40;0 = greatest pain level; 40 = lowest pain level	
19. WOMAC Total Score:0–160;0 = greatest functional impairment; 160 = lowest functional impairment	

### Statistical analysis

Demographic and clinical characteristics for the sample of 140 orthopedic hip patients were described using the sample mean and standard deviation for continuous variables and the frequency and percentage for categorical variables.

To utilize the maximum potential of the data, we followed the recommendations of Schomaker and Heumann and carried out both multiple imputation of missing data and the bootstrap [32]. Starting with the initial pool of 19 variables, a filtering process was used to identify a subset of variables that seemed to be associated with age of onset The process was implemented using the Lasso-penalized variable selection method with the Bayesian information criterion, in the context of a multiple linear regression model for the outcomes of age of onset of DDH and FAIS that was based on 10,000 bootstrap samples [33]. The goal of the LASSO-penalized linear regression was to select a parsimonious and well-fitting subset of variables that associate with age of onset by performing simultaneous variable selection and parameter estimation. This is done by optimizing a penalized least squares criterion that expresses a balance between good fit and parsimony. Moreover, the LASSO variable selection along with the 10,000 bootstrap samples provides sufficient power to estimate and test population parameters. Statistical analyses were carried out using SAS software, version 9.4 (SAS Institute, Inc., Cary, NC, USA). The level of significance was set at α = 0.05 (two-tailed).

## Results

### Participant characteristics

One-hundred and forty patients met inclusion criteria 60% with a diagnosis of FAIS and 40% with a diagnosis of DDH. Of the 84 FAI syndrome patients, 64% were females, the mean age was 36 ± 13 years (age range = 15 to 66 years). The average age of FAI syndrome onset was 33 ± 14 years. Fifty six DDH patients met inclusion criteria, 79% were females, the mean age was 32.12 ± 11.02 years (age range = 14 to 65 years). The average age of DDH onset was 28 ± 12 years. Demographic and clinical characteristics of the 140 patients in the current study are shown in Table 2.

**Table 2:** Demographic and clinical characteristics of patients with symptomatic FAIS and DDH

Characteristic	FAI syndrome (*n* = 84)	DDH (*n* = 56)
Age, years, M (SD)	36.32 (13.02)	32.12 (11.02)
Female Gender, % (n)	64.29% (54)	78.57% (44)
BMI, kg/m^2^, M (SD)	26.32 (6.21)	26.76 (4.83)
BMI > 30 kg/m^2^, % (n)	15.48% (13)	33.93% (19)
BMI ≤30 kg/m^2^, % (n)	84.52% (71)	66.07% (37)
Age of Onset, years, M (SD)	32.95 (13.86)	27.77 (12.03)
Tonnis Grade 1, % (n)	42.86% (36)	32.14% (18)
Tonnis Grade 0, % (n)	57.14% (48)	67.86% (38)
History of Hip Surgery, % (n)	8.33% (7)	12.50% (7)
Lateral Center Edge Angle, M (SD)	31.54 (5.52)	13.89 (7.20)
Alpha Dunn Angle, M (SD)	67.01 (9.79)	62.82 (11.77)
Alpha Frog Angle, M (SD)	63.35 (9.02)	60.55 (10.40)
iHOT (Quality of Life), M (SD)	51.66 (22.23)	41.64 (20.01)
WOMAC Pain, M (SD)	30.19 (17.01)	23.17 (7.09)
SF12 Physical Functioning, M (SD)	42.66 (10.66)	37.75 (11.59)
HADS, M (SD)	10.23 (6.89)	12.26 (6.72)
PCS, M (SD)	13.63 (11.72)	17.35 (11.61)

*M* Sample mean, *SD* Standard deviation, *PCS* Pain catastrophizing scale total score

#### Testing for multicollinearity

To ascertain the presence of any multicollinearity in our LASSO-penalized linear regression models, we examined the Variance Inflation Factor (VIF) for each of the selected variables. The estimated VIFs for the variables were all close to one and less than two, suggesting that multicollinearity was not present or problematic for any of the selected variables in our LASSO-penalized linear regression models.

### Variables associated with age of onset of orthopedic hip disorder

**Fig. 1:**
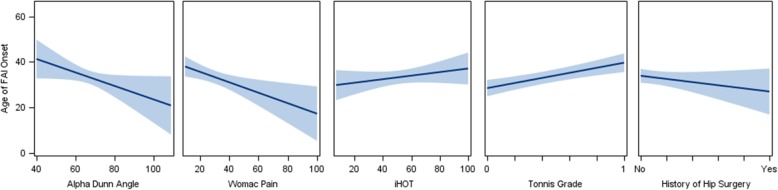
Plot of Age of FAI syndrome Onset (years) against the selected variables, with a fitted linear regression line and 95% confidence limits. Tonnis Grade (grade 1 vs. grade 0) and History of Hip Surgery (Yes vs. No), with the fitted regression line at the mean level of each group. Observed sample, *N* = 84 for the FAI syndrome group

**Fig. 2:**
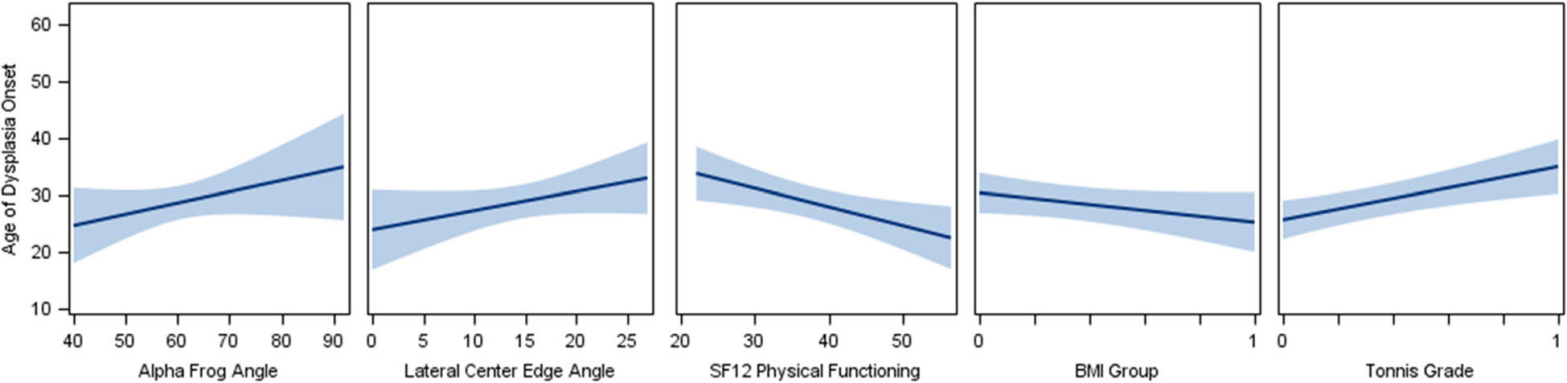
Plot of Age of DDH Onset (years) against the selected variables, with a fitted linear regression line and 95% confidence limits. BMI group (coded 1: > 30 kg/m^2^ vs. coded 0: ≤30 kg/m^2^) and Tonnis Grade (grade 1 vs. grade 0), with the fitted regression line at the mean level of each group. Observed sample, *N* = 56 for the DDH group

The subset of variables that were selected from the LASSO-penalized variable selection method for the outcome of age of onset is reported in Tables 3 and 4 and shown in Figs. 1 and 2. We reported the averaged LASSO-penalized parameter estimates and standard deviation based on 10,000 bootstrap samples of the multiple linear regression models along with the 95% bootstrap confidence interval. For the 95% CI that did not contain zero, the respective mean parameter estimate was statistically significant at alpha = 0.05 (two-tailed). First, we note that pain catastrophizing, anxiety, and depression were not retained in the final model as reported below (for either FAIS or DDH), because these variables did not contribute to the minimization of the final model’s error in determining age of onset. Thus, pain catastrophizing, anxiety, and depression were not significantly related to age of DDH onset (*p*-values > 0.27, *R*^2^ = 0.0213) or age of FAIS onset (*p*-values > 0.29, *R*^2^ = 0.0119).

**Table 3:** Age of FAI syndrome onset

Model Outcome and Variables of Significant Association	Mean Estimate	SD	95% CI	Standardized Estimate	Adjusted R2
Intercept	41.9464	6.7085	30.9471 to 53.6079	0	0.3099
Tonnis Grade (1 vs. 0)	10.8888	2.6368	5.2199 to 15.5958	0.4089	
WOMAC Pain	−0.1669	0.0714	−0.2963 to − 0.0457	− 0.2793	
iHOT (Quality of Life)	0.0926	0.044	0.0263 to 0.1939	0.1784	
History of Hip Surgery (Yes vs. No)	−6.2836	3.1424	−12.7454 to − 1.5595	−0.1354	
Alpha Dunn Angle	−0.1967	0.1076	−0.4009 to − 0.0275	−0.0863

**Table 4:** Age of DDH onset

Model Outcome and Variables of Significant Association	Mean Estimate	SD	95% CI	Standardized Estimate	Adjusted R2
Intercept	16.8407	8.344	− 0.9374 to 31.7791	0	0.3578
Tonnis Grade (1 vs. 0)	5.733	2.8341	1.0041 to 11.2229	0.2395	
SF12 Physical Functioning	−0.2302	0.1086	−0.4739 to − 0.0595	−0.2201	
Alpha Frog Angle	0.2521	0.1187	0.0687 to 0.5245	0.1826	
BMI group (> 30 kg/m2 vs. ≤30 kg/m2)	−4.5927	2.2554	−9.0442 to −1.0147	−0.1763	
Lateral Center Edge Angle	0.2782	0.1428	0.0567 to 0.5795	0.1423

#### FAIS

Alpha angle on Dunn, Tonnis grade, prior hip surgery, WOMAC pain score, and iHOT total score (quality of life) were significantly related to age of onset in FAIS (Adjusted *R*^2^ = 0.3099; Table 3). Age of FAIS onset decreased as alpha Dunn angle increased. Patients with a history of hip surgery were on average 6.28 years younger in their age of FAIS onset than those without a history of hip surgery (95% CI: − 12.74 to − 1.56 years younger). Patients with a Tonnis grade of 1 were on average 10.88 years older in their age of FAI syndrome onset than those with a Tonnis grade of 0 (95% CI: 5.21 to 15.59 years older). Based on standardized parameter estimates (coefficients), the retained variables of significant association with age of FAIS onset are ordered in terms of magnitude of relative importance: Tonnis grade (0.4089), WOMAC pain (standardized coefficient = − 0.2793), iHOT/quality of life (0.1784), history of hip surgery (− 0.1354), and alpha Dunn angle (− 0.0863). Plot of age of FAI syndrome onset against the selected variables, with a fitted linear regression line and 95% confidence limits, is shown in.

#### DDH

As shown in Table 4, lateral center edge angle (LCEA), alpha frog angle, Tonnis grade, SF12 physical functioning, and BMI group were significantly associated with age of DDH onset (Adjusted *R*2 = 0.3578). Age of onset decreased as LCEA and alpha frog angle decreased. Patients with a Tonnis grade of one were on average 5.73 years older in their age of onset than those with a Tonnis grade of zero (95% CI: 1.00 to 11.22 years older). Age of onset decreased as physical functioning (activity level) improved. Patients with a BMI > 30 kg/m2 were on average 4.59 years younger in their age of DDH onset than those with a BMI ≤30 kg/m2 (95% CI: − 9.04 to − 1.01 years younger). Based on standardized parameter estimates (coefficients), the retained variables of significant association with age of DDH onset are ordered in terms of magnitude of relative importance: Tonnis grade (0.2395), SF12 physical functioning (− 0.2201), alpha frog angle (0.1826), BMI group (− 0.1763), and LCEA (0.1422). Plot of age of DDH onset against the selected variables, with a fitted linear regression line and 95% confidence limits, is shown in Fig. 2.

## Discussion

The primary objective of this study was to determine whether pain catastrophizing, anxiety, and depression were associated with age of onset in DDH or FAIS. A secondary aim was to assess whether other radiographic and clinical variables were also related to age of onset in DDH and FAIS. It has been shown that elevated levels of pain catastrophizing, anxiety and depression are significantly present in those with DDH and FAIS [34]. Given the ability to negatively modulate an individual’s perception of pain and worsen outcome, it was predicted that a higher PCS score and HADS score would lead to a younger age of onset in FAI syndrome and DDH. However, in our group of patients, we did not find a correlation with a patient’s perception of pain and catastrophizing and other psychological factors with that of age of onset.

Despite little research establishing the role of mental status in predicting age of onset, many studies have shown that those with worse mental states report greater pain scores, worse surgical outcomes and worse overall well-being [11, 12, 14]. In a study of 396 patients undergoing arthroscopy for FAI syndrome, Westermann et al. found that patient-reported factors, such as mental health, were considered to be more associated with baseline hip pain than articular findings. Moreover, in a study of 301 FAI syndrome patients undergoing arthroscopy, Lansdown et al. found that the presence of a mental disorder correlated with lower patient-reported outcomes before and after surgical treatment [15]. It is likely that individuals with higher PCS and HADS scores will exhibit greater pain scores, but based on our study, they did not affect the age of onset of pain [7, 8].

### FAIS

The population of patients diagnosed with FAIS tend to be young individuals who are active [21, 35]. Activity level, as measured by patient reported questionnaires (i.e UCLA), and severity of disease, as measured by alpha angle on Dunn, have each been shown to be significantly correlated to diagnosis of FAI syndrome [2, 3, 20, 36, 37]. Based on our study, greater radiographic severity and functional impairment of FAIS were associated with younger age of onset.

FAIS is insidious by nature and becomes more symptomatic with increasing severity [3, 38]. The relationship between FAIS and severity of articular damage has been previously studied. For example, Byrd and colleagues showed that in their study of hips surgically treated for FAIS, 98% had a Tonnis grade ≥ 1 and demonstrated articular cartilage damage [39]. In our study, it was determined that those diagnosed with FAIS with a Tonnis grade of 1 had an age of onset 11 years older than those with a Tonnis grade of 0. This aligns with the progression of FAI syndrome and our current understanding of the relationship between age and Tonnis grading. Older age has been associated with greater articular damage, increased risk of arthritis and subsequently higher Tonnis grading [5, 40].

Alpha angle on Dunn radiographs has been evaluated as an important diagnostic measure and indicator of severity in FAIS. Several studies have shown that higher alpha angles are associated with a greater likelihood of FAIS diagnosis and increased severity [2, 36, 37]. In a study evaluating the validity of alpha angle, Barton et al. found that alpha angle on Dunn had 90% rates in sensitivity, positive predictive value and accuracy in the diagnostic evaluation of FAI syndrome [36]. Moreover, Beaule et al. found that in those diagnosed with FAIS, an alpha angle of 65° or greater were found with more severe presentations of cartilage damage [37]. The results of our study agree with these prior studies. Our study is unique in that it allowed for further inspection in the role of alpha angle and its association with age of onset. In our cohort of FAIS patients, increased alpha angle on Dunn were noted on diagnosis, and we found that greater values of alpha angles correlated with younger ages of FAIS onset. Although the magnitude of the effect of alpha angle on Dunn was relatively weak compared to other variables retained in the FAIS model, the significant relationship between alpha angle on Dunn and age of onset provides insight on a variable previously unknown to affect age of onset. Further understanding of alpha angle on Dunn would not only reaffirm its significant role in diagnosing FAIS, but also shed light on its impact on symptomatic presentation of hip pathology patients.

Given the association with activity level and FAIS one would expect UCLA activity to be associated with age of FAIS onset [2, 3, 23]. In fact, Westermann et al. found that patient factors, including activity levels, were more predictive of hip pain than articular findings of FAI syndrome [41]. Despite this, the association between activity level and FAIS was not found in our evaluation of age of FAIS onset. Increasing activity levels (as measured by the UCLA scale) is not associated with younger age of FAIS onset in the current study.

### DDH

Based on our study, greater radiographic severity, high activity level as measured by SF12, and increased BMI were individually associated with an earlier age of onset in DDH. DDH may lead to earlier onset of hip osteoarthritis, and the outcomes of hip preservation techniques such as the Bernese periacetabular osteotomy, are better in younger patients. Wells et al. concluded that patients with ages older than 25 were associated with long term FAI syndrome based on pain scores [42]. In our cohort of DDH patients, a higher Tonnis Grade was associated with an older age of onset. As in FAI syndrome, this reflects the natural progression of DDH and aligns with our current understanding of the relationship between age, cartilage degeneration, and Tonnis grading [43, 44]. In order to maximize success rates of PAO and reduce onset of end stage arthritis, early diagnosis and treatment in the early stages of disease is important.

An important measure of acetabular morphology is LCEA. Decreasing values were associated with a diagnosis of DDH and increasing severity of DDH [6, 20].In fact, several prior studies found that severe DDH, as measured by LCEA, is an independent predictor of younger age at presentation for PAO in symptomatic DDH [6, 45, 46]. A similar conclusion, with respect for age of DDH onset, was made in our study. In our cohort of dysplastic patients, lower LCEA values correlated with younger ages of onset. Despite a relatively weak correlation found, we were able to affirm prior studies that concluded a significant relationship between age of onset and LCEA. LCEA, like alpha angle on Dunn with FAI syndrome, serves as a possible indicator of diagnosis, severity of disease, and age of onset. Research has also found that increasing severity of DDH can lead to increased risk, prevalence, and earlier presentation of end stage arthritis [6, 45, 46]. Our results shed light on the role of radiologic variables such as LCEA and alpha angle on Dunn in helping us understand age of symptomatic onset.

BMI and activity levels play a role in the development of DDH. As previously discussed, the association between DDH and arthritis is significant. Several studies have shown a relationship between higher BMI and activity level, and an increased risk of hip arthritis [47–49]. For instance, in the Melbourne Collaborative project Study of over 38,000 people, those with a higher BMI had an increased risk of total hip replacement [49]. Moreover, Matheney et al. found that patients with higher activity levels presented for arthroplasty of end stage arthritis at significantly younger ages than less active patients [6]. We found that both BMI and activity level, as measured with SF-12 PF, were each found to be associated with age of DDH onset. UCLA was not found to be related to age of DDH onset. Younger ages of onset correlated with higher BMI (> 30 kg/m^2^) and greater activity levels. Modifying activity level and reducing BMI with weight control may delay onset of DDH and minimize risk of arthritis.

## Limitations

This study is not without limitations. Age of onset is dependent on patient reported duration of symptoms at time of presentation. It is likely that this reported duration may be over or underestimated by the patient—making recall bias a possible limitation of this study. However, we note that the concurrent evaluation by the treating orthopaedic surgeon, in part, helps to mitigate this concern. Moreover, all patients were recruited from a large academic tertiary referral center, which may have selected for patients with a higher burden of disease. Lastly, since we took a convenient sample of patients who presented to the clinic, which was not random, this may have led to some survey sampling bias.

## Conclusion

Psychologic factors such as pain catastrophizing, anxiety, and depression were not found to be significantly associated with age of onset in developmental dysplasia of the hip or femoroacetabular impingement syndrome. Despite this, other clinical and radiographic variables were associated with age of onset. For FAIS, functional impairment as measured by WOMAC pain and impaired active lifestyle as measured by iHOT were found to affect age of onset. For DDH, functional impairment as measured by SF12 and increasing BMI were found to be associated with age of onset. Notably, severity of disease was associated with an earlier age of onset in DDH and FAI syndrome. Those with increased severity, as measured by LCEA and alpha Dunn, presented symptomatically at younger ages.

Our study provides insight into the current understanding of age of onset. A radiographically more severe disease does correlate with earlier age of onset in DDH and FAI syndrome, whereas a patient’s perception of pain did not influence the age of onset in hip preservation patients. Psychological factors were not associated with an earlier age of onset in DDH or FAIS. Also, increasing BMI was related on an earlier onset of symptoms in patients with DDH. A patient’s radiographic severity may have more of a relationship to the onset of pain than physiologic factors.

## Data Availability

The datasets generated and/or analyzed during the current study are not publicly available due concern for patient privacy but are available from the corresponding author on reasonable request.

## Abbreviations

DDH: Developmental dysplasia of the hip
FAIS: Femoroacetabular impingement syndrome
HADS: Hospital and Anxiety Depression Score
LCEA: Lateral center edge angle
PAO: Periacetabular osteotomy
PCS: Pain catastrophizing scale
